# Prevalence, incidence and correlates of HSV-2 infection in an HIV incidence adolescent and adult cohort study in western Kenya

**DOI:** 10.1371/journal.pone.0178907

**Published:** 2017-06-06

**Authors:** Brenda Akinyi, Collins Odhiambo, Fredrick Otieno, Seth Inzaule, Simon Oswago, Emily Kerubo, Richard Ndivo, Clement Zeh

**Affiliations:** 1 Centre for Global Health Research, Kenya Medical Research Institute (KEMRI), Kisumu, Kenya; 2 Division of HIV/AIDS Prevention, Centers for Disease Control and Prevention (CDC), Kisumu, Kenya; Southern Illinois University School of Medicine, UNITED STATES

## Abstract

**Background:**

Herpes simplex virus type 2 (HSV-2) infections are associated with increased risk of HIV transmission. We determined HSV-2 prevalence, incidence and associated risk factors, incidence among persons with indeterminate results, and prevalence of HSV-2/HIV co-infection among young adults (18–34 years) and adolescents (16–17 years) enrolled in an HIV incidence cohort study in western Kenya.

**Methods:**

Participants (n = 1106; 846 adults) were screened and those HIV-1 negative were enrolled and followed-up quarterly for one year. HSV-2 was assessed using the Kalon enzyme immunoassay. HSV-2 incidence was calculated separately among HSV-2 seronegative participants and those indeterminate at baseline. Logistic regression was used to estimate the odds of HSV-2 infection and Poisson regression was used to assess HSV-2 incidence and associated factors.

**Results:**

Overall, HSV-2 prevalence was 26.6% [95% confidence interval (CI): 23.9–29.4] and was higher in adults (31.5% [95% CI: 28.3–34.9]) than adolescents (10.7% [95% CI: 7.1–15.3]). Factors associated with prevalent HSV-2 included female gender, increasing age, HIV infection, history of sexually transmitted infection, low level of education, multiple sexual partners, and being married, divorced, separated or widowed. Overall HSV-2 incidence was 4.0 per 100 person-years (/100PY) 95% CI: 2.7–6.1 and was higher in adults (4.5/100PY) and females (5.1/100PY). In multivariable analysis only marital status was associated with HSV-2 incidence. Among 45 participants with indeterminate HSV-2 results at baseline, 22 seroconverted, resulting in an incidence rate of 53.2 /100PY [95% CI: 35.1–80.9]. Inclusion of indeterminate results almost doubled the overall incidence rate to 7.8 /100 PY [95% CI: 5.9–10.5]. Prevalence of HIV/HSV-2 co-infection was higher in female adults than female adolescents (17.1 [95% CI: 13.6–21.0] versus 3.4 [95% CI: 1.1–7.8]).

**Conclusion:**

The high incidence rate among persons with indeterminate results underscores the public health concerns for HSV-2 spread and underreporting of the HSV-2 burden. Careful consideration is needed when interpreting HSV-2 serology results in these settings.

## Introduction

Herpes simplex virus type 2 (HSV-2) infections are a leading cause of genital ulcer disease [1, 2]. Global burden of HSV-2 is about 11.3%, but prevalence varies in different regions, with sub-Saharan Africa accounting for 31.5% of all infections worldwide [3]. Although HSV-2 infection generally has asymptomatic periods, it is capable of causing significant morbidity, occurring mainly as ulcerative infections of the genital mucosa with painful lesions that may be recurrent. Therefore, management of genital HSV-2 infection should address the chronic nature of the disease rather than focusing solely on treatment of acute episodes of genital lesions [4]. In addition, HSV-2 can be passed from mother to child during childbirth, causing serious neonatal infections, which are usually fatal in the absence of treatment. Moreover, HSV-2 infection has been linked to HIV infection, and it is believed to increase susceptibility to HIV infection two to three fold and transmission of HIV infection by up to five fold. In fact, nearly 50% of HIV infections in populations with at least 80% HSV-2 prevalence are attributed to HSV-2 infections [5].

Because HSV-2 is chronic, periodically symptomatic, and capable of causing long-term morbidity along with the risk of HIV acquisition [5], it is a significant public health concern, which needs active prevention strategies, especially among the most at-risk populations [6]. Various studies have evaluated the risk factors for HSV-2 infection among high-risk groups but few have been conducted in the general population, especially among adolescents. The identification of risk factors is vital for prevention purposes, especially in areas at high risk of HIV acquisition. Moreover, since HIV shares common risks with HSV-2, addressing the risk factors of HSV-2 is likely to also reduce HIV infection rates. It is known that male circumcision significantly reduces both HSV-2 and HIV infection [7]. This risk reduction is thought to be mediated by a reduction in HSV-2-associated genital ulcers. Other identified risk factors for HSV-2 include female gender[3], multiple sexual partners, young age of sexual debut, and previous history of sexual transmitted infections (STI) [4].

HSV-2 generally remains undiagnosed despite its high burden. This is because of its long sub-clinical phase with varied and non-specific clinical manifestations. Most treatment guidelines are not specific or clear when referring to asymptomatic HSV-2 infections or indeterminate diagnosis. This lack of guideline specificity may limit appropriate preventive and treatment efforts; hence, confirmatory laboratory testing is vital in HSV-2 diagnosis and management. Commonly, HSV-2 testing is performed using serological enzyme-linked immunoassay (ELISA) tests. A challenge with these assays is the inconclusive diagnosis due to indeterminate results. Some studies have suggested raising the assay index value, especially in high HSV-2 prevalence areas [8]. Most studies however, exclude the indeterminate results [9] since there are no clear guidelines for management or therapy. This practice may create a biased estimate of HSV-2 burden and also deny the opportunity for early or suppressive treatment and prevention efforts at the individual level. Our objectives were to determine HSV-2 prevalence, HSV-2 incidence, correlates of HSV-2 prevalence and incidence, HSV-2 incidence among persons with indeterminate results, and prevalence of HSV-2 and HIV co-infection in an HIV-1-at-risk and infected adult and adolescent population in western Kenya.

## Materials and methods

### Study population

Between January 2007 and June 2010, 1,106 adolescents (16–17 years) and young adults (18–34 years) were screened for enrolment into the Kisumu Incidence Cohort Study (KICoS) from the catchment area of Kisumu, in western Kenya [10]. The study procedures and methods for screening, collection of demographics, behavioral and clinical information, HIV testing, and quarterly follow-up have been previously described in detail [10]. Briefly, community engagement occurred prior to initiating study recruitment in Kisumu and the surrounding area (within 150 kilometres of the city). Convenience sampling was used to recruit study participants given that the parent study was designed to identify persons willing to participate in a prospective study. At the baseline visit, we collected information on current and previous STIs, sexual behavior, and socio-demographic characteristics, using staff-administered computer-assisted personal interview (CAPI) and participant self-administered audio computer-assisted self-interview (ACASI). In addition, participants underwent a medical examination and laboratory testing for STIs, including HSV-2, regardless of symptoms. Rapid HIV testing was also provided after counselling and those infected were referred for HIV care and treatment per the national guidelines. Circumcision status was based on participant self-report. Laboratory results were returned within two weeks and participants who tested positive for one or more STI were treated and counselled.

Participants who reported having sexual intercourse at least once in the past three months and were HIV-uninfected were enrolled and followed for a period of one year to determine HIV incidence. Follow-up visits were conducted at the clinic every three months where data were collected and blood drawn for HSV-2 and HIV testing. For those with symptoms, STIs were tested and treated during the quarterly visits.

### HSV-2 laboratory testing

Whole blood was collected from the study participants and taken to the ISO 15189 accredited Kenya Medical Research Institute’s (KEMRI) HIV- Research laboratory collaborating with the US Centers for Disease Control and Prevention (CDC), for serum separation and testing of HSV-2. The HSV-2 IgG ELISA (Kalon Biological Limited, Guildford, UK) was performed on all serum samples for the detection of IgG antibodies to HSV-2, with an adjusted cut-off of 2.0. An outcome was considered positive if the optical density (OD) ≥ 2.0, negative if OD< cut-off 0.9 and indeterminate if cut-off 0.9≤sample OD < cut-off 2.0. Repeat testing was performed on all positive or indeterminate samples along with 5% of randomly selected negative samples per manufacturer’s recommendations as a quality assurance measure. HIV serostatus was determined by a parallel testing algorithm of two enzyme immunoassays, at screening and prospectively at each three-month interval visit, during the 12 months of follow-up.

### Ethical review

This study was approved by the scientific steering and ethics review committees of KEMRI (protocol no. SSC 1125), and the Institutional Review Board of the CDC (protocol no. 4938). All participants provided signed informed consent with assent obtained in addition to parental/guardian consent for minors. All persons who took part in the eligibility screening and follow-up visits received a standard transport reimbursement of KES 300 (USD 3.00). In addition, participants received counselling and treatment for STIs and other common ailments and were given condoms (men and women).

### Statistical methods

Correlates of HSV-2 at baseline were assessed by use of sociodemographic and behavioral covariates using logistic regression analysis. We assessed associations between HSV-2 incidence with fixed covariates such as gender, age, marital status which were reported at enrolment; time-varying covariates such as injections and drug use in the last three months which were reported during follow-up visits. Variables with *P* < 0.25 were entered into a multivariable model to adjust for potential confounders.

The HSV-2 incidence rate was estimated using a generalized linear mixed model assuming a Poisson distribution and using a log link function. This was performed using three methods: 1) on samples negative at baseline and positive at one year follow-up (confirmed HSV-2 infected), 2) on the indeterminate samples at baseline and HSV-2 positive at one year follow-up, and 3) on the indeterminate samples at baseline and HSV-2 positive or indeterminate at one year. Infection was assumed to have occurred at the mid-time point between the last visit with negative and first visit with a positive serological test. Incidence was estimated per 100 person years (PY). Adjusted incidence rate ratios (adjIRRs) were estimated for fixed and time-varying covariates (to account for changes in risk behaviours over time) using a Poisson model. Exact binomial CIs were calculated for the prevalence estimates. Records with missing data were dropped in the regression analysis.

## Results

A total of 1,106 participants were screened, of whom 846 were adults and 260 were adolescents. Of the 1,106 participants screened, 831 HIV-seronegative persons met the inclusion criteria and were enrolled into the study; 625 adults (75.2%) and 206 (24.8%) adolescents. Twelve participants, 8 adults and 4 adolescents, were HIV-infected during follow up, 5 of whom were HSV-2 seropositive at baseline.

### HSV-2 prevalence

Of 1,106 participants at screening, one participant had no HSV-2 result while 81 had indeterminate HSV-2 results and were excluded from the analysis. Of 1,024 participants included in the analysis, 492 (48.0%) were males and 243 (23.7) were adolescents. Overall, HSV-2 prevalence was 26.6% (95% confidence interval [CI] 23.9% - 29.4) and was higher in adults than adolescents; 31.5% (95% CI [28.3–34.9] and 10.7% CI [7.1–15.3] respectively (Table 1). Females generally had a higher HSV-2 prevalence than males, with a prevalence ratio of 3.4 (95% CI [2.6–4.4]; p<0.001) among adults and 9.0 (95% CI 2.1–37.1; p = 0.002) among adolescents (Table 1).

**Table 1:** Prevalence of HSV-2 at enrolment, by gender and age-group, Kisumu Incidence Cohort Study, Kisumu, Kenya, 2007–2010.

Population	HSV-2 Prevalence			
	N (%)	95% CI	PR [95% CI]	*p-value*
Overall	272 (26.6)	23.9–29.4	** **	
Adults	246 (31.5)	28.3–34.9		*<0*.*001*
Adolescents	26 (10.7)	7.1–15.3		*<0*.*001*
Adult^a^ Males	56 (14.4)	11.1–18.3	*ref*.	
Adult^a^ Females	190 (48.4)	43.3–53.4	3.4 [2.6–4.4]	< 0.001
Adolescent^b^ Males	2 (1.9)	0.2–6.8	*ref*.	
Adolescent^b^ Females	24 (17.3)	11.4–24.6	9.0 [2.1–37.1]	0.002

Abbreviations: HSV: Herpes Simplex Virus, CI: Confidence Interval, PR: Prevalance Ratio

Definitions

^a^Adults 18–34 years

^b^Adolescents 16–17 years

### Correlates of HSV-2 prevalence

HSV-2 prevalence increased with age; those aged 18–24, 25–29 and 30–34 years had prevalence of 26.7%, 40.0% and 62.5% respectively. Prevalence was also higher in females (40.2%) compared to male (11.8%), HIV-infected (75.2%) compared to HIV-uninfected (20.3%), married (48.5%) or separated/divorced/widowed (62.2%) compared to single/never married (15.7%), circumcised males (13.7%) compared to uncircumcised males (10.9%), and those with previous STI (42.2%) compared to those never treated for an STI (24.0%) (Table 2).

**Table 2:** Factors associated with HSV-2 prevalence at enrolment, among participants in Kisumu Incidence Cohort Study, Kisumu, Kenya 2007–2010.

Characteristics		HSV-2 infected N (%)	Unadjusted OR (95% CI)	p-value	Adjusted OR (95% CI)	p-value
**Overall prevalence**		272 (24.6)				
**Age**	**16–17 years**	26 (10.7)	*Ref*	<0.001	Ref	<0.001
	**18–24 years**	159 (26.7)	3.0 (2.0–4.8)	<0.001	2.8 (1.6–5.0)	<0.001
	**25–29 years**	52 (40.0)	5.6 (3.3–9.5)	<0.001	3.6 (1.8–7.5)	<0.001
	**30–34 years**	35 (62.5)	14.0 (7.1–27.4)	<0.001	7.9 (3.0–21.0)	<0.001
**Gender**	**Male**	58 (11.8)	*Ref*		*Ref*	
	**Female**	214 (40.2)	5.0 (3.6–7.0)	<0.001	7.3 (4.7–11.5)	<0.001
**HIV Status**	**Uninfected**	184 (20.3)	Ref		Ref	
	**Infected**	88 (75.2)	11.9 (7.6–18.7)	<0.001	5.6 (3.2–9.8)	<0.001
**Education**	**Technical training/College**	39 (14.3)	*Ref*		*Ref*	
	**Secondary**	101 (28.7)	2.4 (1.6–3.6)	<0.001	2.6 (1.6–4.2)	<0.001
	**Primary**	114 (32.4)	2.9 (1.9–4.3)	<0.001	2.8 (1.6–4.8)	<0.001
	**Never attended school**	16 (39.0)	3.8 (1.9–7.8)	0.001	2.9 (1.1–7.6)	0.027
**Marital Status**	**Single/Never married**	110 (15.7)	*Ref*		*Ref*	
	**Married/living as married**	131 (48.5)	5.1 (3.7–6.9)	<0.001	2.5 (1.6–3.9)	<0.001
	**Separated/Divorced/Widowed**	28 (62.2)	8.9 (4.7–16.7)	<0.001	3.4 (1.4–8.2)	0.008
**Circumcision Status#**	**Circumcised**	26 (13.7)	*Ref*			
	**Uncircumcised**	32 (10.9)	0.8 (0.4–1.3)	0.348	* *	
**Ever treated for STI**	**No**	213 (24.0)	*Ref*	* *	*Ref*	
	**Yes**	54 (42.2)	2.3 (1.6–3.4)	<0.001	2.5 (1.5–4.2)	0.001
**Lifetime number of sexual partners**	**0–1 partner**	40 (22.5)	*Ref*		*Ref*	
	**2–3 partners**	104 (30.0)	1.5 (1.0–2.3)	0.069	2.0 (1.2–3.4)	0.014
	**≥ 4 partners**	118 (26.4)	1.2 (0.8–1.9)	0.309	2.1 (1.2–3.7)	0.008

#Applicable to male participants only

Abbreviations: HSV: Herpes Simplex Virus, CI: Confidence Interval, STI: Sexually transmitted infection, OR: Odds ratio

Definitions: Adults 18–34 years; Adolescents 16–17 years

**Fig 1:**
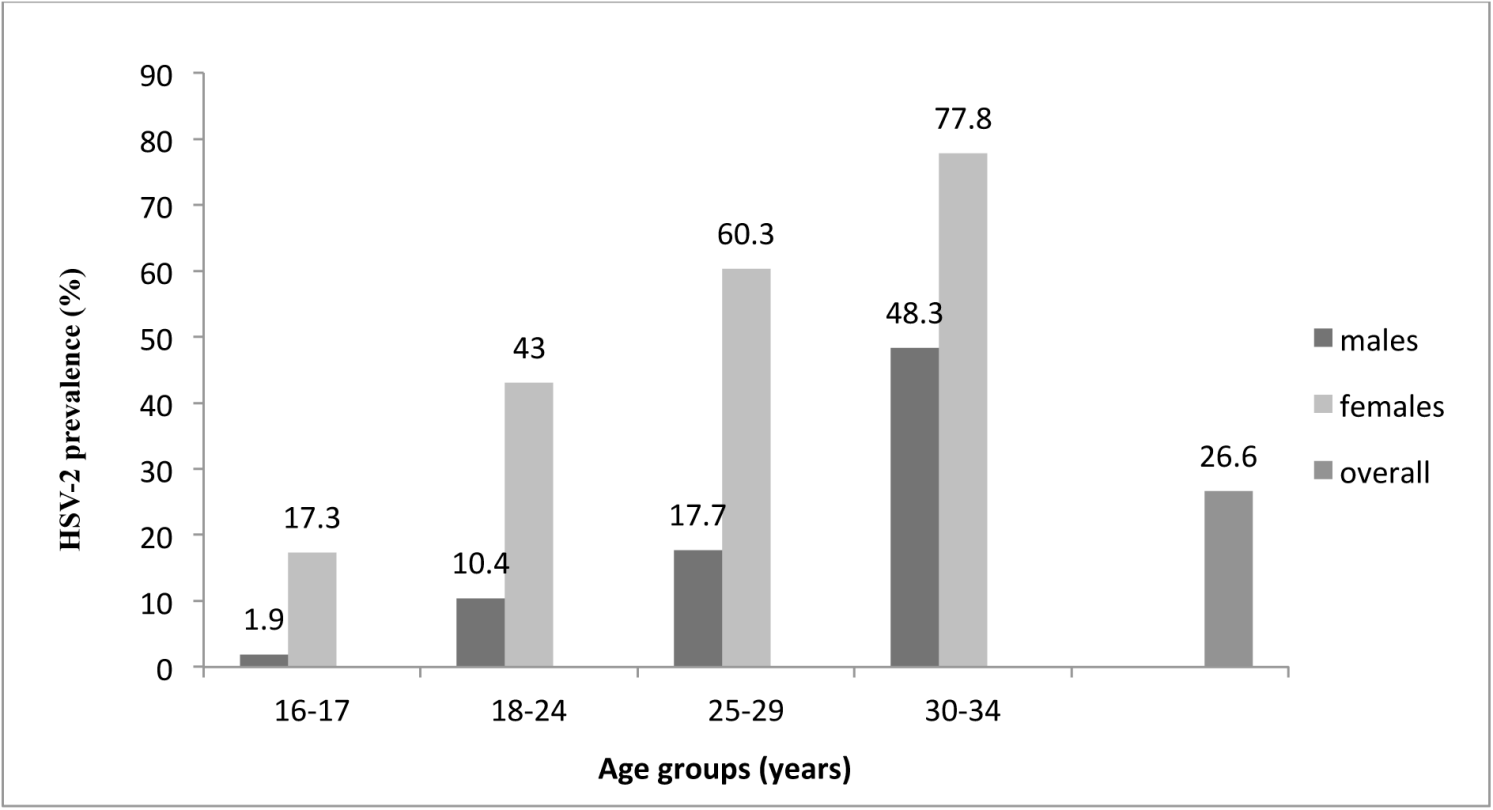
HSV-2 prevalence at enrolment, stratified by age group and gender, among participants in the Kisumu Incidence Cohort Study, Kisumu, Kenya 2007–2010.

In the multivariable model, the odds of having a prevalent HSV-2 infection were higher among adult participants compared to adolescents (16–17 years), with odds increasing with age. The adjusted ORs (aOR) were 2.8 (95% CI 1.6–5.0), 3.6 (95% CI 1.8–7.5), and 7.9 (95% CI 3.0–21.0) for participants in the 18–24, 25–29, and 30–34 age groups, respectively. The odds of having a prevalent HSV-2 infection were significantly associated with being female (aOR; 7.3, 95% CI 4.7–11.5), HIV-infected (aOR 5.6 95% CI 3.2–9.8), having a lack of formal education (aOR 2.9, 95% CI 1.1–7.6) compared to those with college education, and married/cohabiting (aOR 2.5, 95% CI 1.6–3.9) or separated/widowed/divorced (aOR 3.4, 95% CI 1.4–8.2) compared to being single/never married. Additionally, HSV-2 prevalence was associated with a history of treated STIs (aOR 2.5, 95% CI 1.5–4.2) and having four or more sexual partners (aOR 2.1, 95% CI 1.2–3.7) (Table 2). HSV-2 prevalence increased with age for both men and women, but the rate was always higher in the women (Fig 1).

### HSV-2 incidence

Of 831 HIV-1-negative participants enrolled into the study, 157 were HSV-2 positive, 57 had indeterminate results. One participant had missing results and was excluded from the incidence analysis, leaving a total of 616 participants. The overall incidence of HSV-2 was estimated at 4.0 (95% CI: 2.7–6.0) /100 PY (Table 3) for participants testing negative at enrolment and followed through the twelve months of study. The incidence was higher in adults than adolescents [(4.5, 95% CI: 2.8–7.1 versus 3.0 95% CI: 1.2–7.1)/100 PY, respectively] and females had higher HSV-2 incidence than males (5.1, 95% CI: 2.8–9.3 versus 3.4, 95% CI: 1.9–6.0)/100 PY, respectively (Table 3).

**Table 3:** HSV-2 incidence rates among HSV-2-negative participants at baseline compared to participants with HSV-2-negative/indeterminate results at baseline during follow up, by age-group and gender, Kisumu Incidence Cohort Study, Kisumu, Kenya, 2007–2010.

	HSV-2 incidence among HSV-2 negative participants at baseline			HSV-2 incidence among participants with HSV-2 negative/Indeterminate results at baseline			
	N	Incidence/100PY	95% CI	N	Incidence/100PY	95% CI	RR [95%CI]
**Cohort**							
Adults	440	4.5	2.8–7.1	484	9.0	6.6–12.4	2.8(1.2–8.0)
Adolescents	176	3.0	1.2–7.1	189	3.3	1.5–7.3	ref
**Gender**							
Male	370	3.4	1.9–6.0	402	9.8	6.5–14.7	1.7(0.9–3.2)
Female	246	5.1	2.8–9.3	271	5.8	2.8–9.3	ref
**Overall**	616	4.0	2.7–6.0	673	7.3	5,5–9.8	7.3(5.5–9.8)

Abbreviations: HSV: Herpes Simplex Virus, CI: Confidence Interval, STI: Sexually transmitted infection, RR: Risk ratio, PY: Person years

Definitions: Adults 18–34 years; Adolescents 16–17 years

### Correlates of HSV-2 incidence

The only factor associated with HSV-2 incidence in the multivariable analysis was being married or cohabiting (risk ratio (RR) 2.8, 95% CI 1.5–5.5) (Table 4).

**Table 4:** Factors associated with HSV2 incidence among participants in Kisumu Incidence Cohort Study, Kisumu, Kenya, 2007–2010.

Characteristics		Unadjusted RR (95% CI)	p-values	Adjusted RR (95% CI)	p-values
**Age group**	16–17 years	* ref*.		* ref*.	
	18–24 years	2.4 (1.0–5.8)	0.047	2.1 (0.9–5.2)	
	25–29 years	3.2 (1.1–9.5)	0.032	2.3 (0.7–7.0)	
	30–34 years	3.2 (0.6–6.3)	0.156	1.8 (0.3–10.1)	0.306
**Education**	Never attended school	3.4 (0.7–16.5)	0.132		
	Primary	2.0 (0.9–4.7)	0.090		
	Secondary	1.6 (0.7–3.9)	0.255		
	Technical training/College	*ref*.			
**Marital Status**	Single/Never married	* ref*.		* ref*.	
	Married/living as married	1.84 (0.3–2.8)	0.545	2.8 (1.5–5.5)	0.040
	Separated/Divorced/Widowed	1.9 (0.3–3.7)	0.542	1.8 (0.3–12.8)	
**Gender**	Male	*ref*.	* *	*ref*.	
	Female	1.8 (1.0–3.3)	0.044	1.7 (0.9–3.2)	0.125
**Had Forced sex in the last 3 months**	Yes	*ref*.			
	No	1.0 (0.3–3.3)	0.966		
**Ever had anal sex**	Yes	*ref*.			
	No	0.7 (0.3–1.6)	0.415		
**Had an injection in the last 3 months**	Yes	1.7 (0.9–1.1)	0.124	1.7 (0.9–3.3)	0.179
	No	*Ref*.	* *	*ref*.	
**Ever had a scarification**	Yes	*ref*.			
	No	1.4 (0.5–4.6)	0.540		
**Ever had contact with someone else's blood**	Yes	*ref*.			
	No	0.8 (0.3–2.5)	0.705		
**Ever treated for STI**	Yes	1.5 (0.4–6.4)	0.552		
	No	*ref*.			
**Ever had genital ulcers**	Yes	13.0 (1.9–87.3)	0.008	11.9 (1.8–79.2)	0.353
	No	*ref*.	* *	*ref*.	

Abbreviations: HSV: Herpes Simplex Virus, CI: Confidence Interval, STI: Sexually transmitted infection, RR: Risk ratio

Definitions: Adults 18–34 years; Adolescents 16–17 years

### HSV-2 incidence among persons with indeterminate results

Based on the inclusion criteria, 24 persons with indeterminate results at baseline were excluded from the incidence analysis leaving a total of 57 participants. Of 57 participants with indeterminate HSV-2 results at enrolment, 22 tested positive by the end of the 12-month follow-up period; while, 23 remained indeterminate, 11 tested negative, and 1 had a missing result. In calculating the incidence only among participants with indeterminate results, the 11 participants with HSV-2 negative results were excluded. Among the 45 persons with an indeterminate result, incidence was high; 53.2 (95% CI 35.1–80.9)/100 PY (Table 5).

**Table 5:** Estimated HSV-2 seroconversion rates by gender and age for HSV-2-indeterminate status participants who seroconverted during 12 months of follow-up. Kisumu Incidence Cohort Study, Kisumu, Kenya, 2007–2010.

	HSV-2 positive (N)	Incidence per 100PY	95% CI
**Gender**			
Male	12	67.0	38.1–118.0
Female	10	42.7	23.0–79.4
**Cohort**			
Adults	21	72.7	47.7–111.6
Adolescents	1	8.0	1.1–57.0
**Total**	22	53.2	35.1–80.9

Abbreviations: HSV: Herpes Simplex Virus, CI: Confidence Interval

Definitions: Adults 18–34 years; Adolescents 16–17 years

When all participants with indeterminate results (N = 57) at screening were included in the incidence analysis, the overall incidence increased to 7.3 [95% CI 5.5–9.8] (Table 3) from 4.0 [95% CI 2.7–6.0] /100PY. Incidence varied by age-group and gender; adults (vs adolescents) had a risk ratio of 2.8 (95% CI 1.2–8.0) *P* = 0.0116, and males (vs females), 1.7 (95% CI 0.9–3.2) *P* = 0.0851 (Table 3).

### Prevalence of HSV-2 and HIV Co-infection

Prevalence of HIV/HSV-2 co-infection *at enrolment* was higher in female adults and adolescents, 17.1 [CI 13.6–21.0] and 3.4 [CI 1.1–7.8] respectively compared to adult and adolescent males, 2.9 [CI 1.5–5.0] and 0 [CI 0–3.2] respectively (Table 6). Five of 157 participants (3.2%) who were HSV-2 seropositive and 7 participants (1.0%) of who were HSV-2 seronegative or indeterminate at baseline acquired HIV infection during follow up.

**Table 6:** Estimated HSV-2/ HIV prevalence rates at enrolment by age-group and gender. Kisumu Incidence Cohort Study, Kisumu, Kenya, 2007–2010

Population	N	HSV-2 co-infection with HIV		Overall HSV-2/HIV Prevalence [Exact 95% CI]
		Prevalence (%) [95% CI]	Odds Ratio [95% CI]	
Adult Male	12	2.9 [1.5–5.0]	*ref*.	
Adult Female	71	17.1 [13.6–21.0]	6.9 [3.6–14.2]	
Adolescent Male	0	0 [0–3.2]	*ref*.	
Adolescent Female	5	3.4 [1.1–7.8]	{undefined}	
Adults				10.0 [8.0–12.2]
Adolescents				1.9 [0.6–4.5]

Abbreviations: HSV: Herpes Simplex Virus, CI: Confidence Interval, OR: Odds ratio

Definitions: Adults 18–34 years; Adolescents 16–17 years

## Discussion

Overall, HSV-2 prevalence for this cohort was 26.6% and incidence was 4.0 /100PY. HSV-2 prevalence was higher among women when compared to men in all age groups in the study. Age-specific prevalence for women in this study was at least one and a half times as high as the national prevalence. (40.2% vs 26.1% respectively) [11] In contrast, the prevalence in males was much lower than the national prevalence (11.8% vs 26.1%). In our analysis, the observed low prevalence for males compared to females, especially among the adolescents, is similar to what has been reported for other STIs, including HIV, and may reflect age-mixing sexual practices in the region [12]. Prevalence rates were lower for both adults and adolescents in all age groups in the current study when compared to a 2009 study among a rural population within the same region. [12, 13].

In our study, HSV-2 prevalance was associated with several factors. The increasing prevalence with age, female gender, low level of education, number of lifetime sexual partners, HIV co-infection, history of sexually transmitted infections, and currently or previously married have also been reported by other studies in this region as HSV-2 associated factors [6, 10, 14]. The high HIV/HSV-2 co-infection observed in this study corroborates existing evidence of synergistic relationship between the two diseases. A proposed mechanism for the synergy includes a high risk of HIV acquisition in persons with HSV-2 due to an influx of HIV target cells in response to HSV-2 replication in the genital mucosa. Conversely, the low immunity associated with HIV infection is thought to increase suspectibility to HSV-2 infection. Therefore, either of the viral infections augments infectivity of the other [14]. Moreover, persons with both infections are equally at risk of transmitting either of the viruses. This suggests that preventive efforts for either viral infection are likely to lead to a reduction in the other infection. Indeed HIV infection in this region follows a similar epidemiological pattern as that of HSV-2 with infections in women increasing as age increases [12, 14].HIV surveys in Kenya have reported a high prevalence of HSV-2 among currently and previously married persons [14]. This association continues to suggest shared risk factors among the two viral infections [15]. However, this could also indicate an increased risk of HIV acquisition from an HSV-2 infected partner or transmission by an HIV/HSV-2 coinfected index case due to repeated contact among discordant couples. While previous clinical trials have failed to show a reduction in risk of HIV acquisition in HSV-2 partners treated with acyclovir suppresive therapy [16, 17], some trials have shown reduction in risk of transmission of HIV by the index case [16]. The low transmission risk is mediated by a reduction in HIV RNA by 0.26–0.53 log_10_, as has been reported in several clinical trials [16]. While the exact biological mechanism of HSV-2 and HIV synergy is not completely understood, *in vitro* studies have shown the activation of HIV-1 target cells by genital herpes for HIV-1, as well as binding and transactivation of the long terminal repeat region of HIV-1, subsequently increases HIV replication. Therefore, HSV-2 control and prevention could play a vital role in HIV-1 reduction in married couples specifally among HIV or HSV-2 discordant couples.The association of HSV-2 with a history of STIs may reflect a behavioral risk rather than biological. As has been reported in other studies, there was an association between number of lifetime sexual partners and HSV-2 infection[14]. The association reflects the need for enhanced behavioural prevention efforts including risk-reduction counselling for those engaging in multiple sexual partnerships.

In this study, the observed HSV-2 incidence was lower than what has been reported in other studies in the region, especially among females. The male HSV-2 incidence was closer to other areas such as Rakai in Uganda, which reported an incidence of 4.9 per 100py [18].

A key finding in this analysis was the greater than half of participants with HSV-2 indeterminate results at baseline seroconverting, resulting in an incidence almost twice that of participants who were HSV-2-negative at baseline. Exclusion of indeterminate HSV-2 results in incidence analysis calculations could have biased the observed incidence. Indeterminate infection is thought to be a result of increased cross-reactivity of HSV-2 antibodies with those of other pathogens, or a reduced sensitivity of the Kalon HSV-2 IgG assay in detecting infections during the seroconversion window. Our findings thus suggest the need for algorithms with a higher index value for the screening tests to increase the sensitivity, especially among the indeterminate results [12, 19]. Alternatively, indeterminate sera need further analysis using a western blot assay to determine if the indeterminate have evidence of other HSV-2 specific bands that appear earlier than IgG2. This is informed by uncertain on whether participants in the indeterminate cohort are really HSV-2 seropositive and/or just starting to seroconvert because IgG2 is a relatively late antibody acquisition and hence the follow-up sera may be a reactivation rather than incident disease.

There were several limitation to our study. A key limitation is the low number of new HSV-2 and exclusion of HIV-1 infections during the 12-month study period. This restricted the ability to conclusively assess risk factors associated with new HSV-2 infections. Also, we used convenience sampling method in our recruitment, which is likely to have introduced selection bias. Additionally, because of the few responses to the circumcision question in our study, we were unable to fully determine its true effect in prevention of HSV-2 infection, as shown in other studies [7, 20]. Moreover, circumcision status was self-reported, hence we were unable to verify the true status of participants who provided a response. Two covariates; forced sex and anal sex had the most missing records in analysis of HSV-2 incidence with up to 280 records missing from a total of 2300 records. This may have been related to the secrecy of sexual encounters among this population. Finally, because we did not know if indeterminate results were negative or positive at baseline, we could not ascertain whether the incidence was too high or too low. However, despite these limitations, our results shed light on the need to re-evaluate HSV-2 algorithms to improve sensitivity in detecting potential seroconverters.

In conclusion, our study highlights the need for prevention efforts of HSV-2 infections, especially among adolescent girls, women, and married couples. With women being disproportionately affected, there is an urgent need for HSV-2 control and prevention efforts targeting adolescent girls and women in this population. The high rate of seroconversion of persons testing indeterminate at baseline, and the resulting increased HSV-2 incidence, underscores the potential public health concerns for HSV-2 spread, as well as the underreporting of HSV-2 burden. Management of genital HSV-2 infections should take into consideration if/when to treat persons with indeterminate results, who are generally asymptomatic, rather than focusing solely on treatment of those symptomatic with genital lesions. There is a need to re-consider the cut-off for interpretation of HSV-2 results by the Kalon serological assay to increase sensitivity and specificity for identification of persons acutely infected with HSV-2.
